# Molecular evidence of *Enterocytozoon bieneusi* in arid urban landscapes of shiraz cockroaches (Blattodea), Southwest Iran: Implications for urban public health surveillance

**DOI:** 10.1016/j.parepi.2025.e00446

**Published:** 2025-07-01

**Authors:** Mohsen Kalantari, Kourosh Azizi, Negin Kiani Junaghani, Mozaffar Vahedi, Iraj Mohammadpour, Qasem Asgari, Amin Hosseinpour, Mehdi Miri

**Affiliations:** a Research Center for Health Sciences, Institute of Health, Department of Biology and Control of Disease Vectors, School of Health, Shiraz University of Medical Sciences, Shiraz, Iran; b Student Research Committee, Department of Biology and Control of Disease Vectors, School of Health, Shiraz University of Medical Sciences, Shiraz, Iran; c Department of Medical Parasitology and Mycology, School of Medicine, Shiraz University of Medical Sciences, Shiraz, Iran

**Keywords:** *Enterocytozoon bieneusi*, *Encephalitozoon intestinalis*, Microsporidia, *Blattodea*, PCR, Iran

## Abstract

Microsporidia, particularly *Enterocytozoon bieneusi* and *Encephalitozoon intestinalis*, are emerging fungal pathogens of global concern, causing severe gastrointestinal and systemic infections in immunocompromised individuals. Cockroaches (Blattodea), as synanthropic pests, are potential mechanical vectors for disseminating these pathogens in urban environments. Despite their clinical significance, data on the role of cockroaches in transmitting microsporidia in arid regions remain scarce. This study assessed the contamination of cockroaches in Shiraz, Iran, with *E. bieneusi* and *E. intestinalis* to evaluate their public health risks. From January to June 2023, 378 cockroaches were collected from high-risk areas, including Hospitals and adjacent public areas. Specimens were morphologically identified and screened via microscopy. Molecular detection of *E. bieneusi* and *E. intestinalis* was performed using PCR targeting the 18sRNA region. DNA was extracted from pooled samples (10 cockroaches/pool) and amplified under standardized thermocycling conditions. All cockroaches were identified as *Periplaneta americana* (77.5 %) and *Blattella germanica* (22.5 %). In molecular assay, three positive case of *E. bieneusi* was detected, However, no *E. intestinalis* DNA was identified via PCR. This study reports the first molecular detection of *E. bieneusi* in Shiraz cockroaches. In continue, it records no evidence of *E. intestinalis*, which suggesting climatic or environmental barriers to limit this microsporidian persistence in this arid region. These findings highlight the need for integrated vector management and advanced molecular surveillance to elucidate the epidemiology of understudied pathogens like microsporidia in rapidly urbanizing regions.

## Highlights

• **First molecular detection** of *Enterocytozoon bieneusi* in cockroaches from Shiraz, Iran, underscoring their role as potential urban vectors.
• **Absence of *Encephalitozoon intestinalis*** in Shiraz contrasts with tropical regions, suggesting climate-driven barriers to microsporidian persistence.
• **Hospital-associated cockroaches** harbored parasite cysts, highlighting risks in healthcare settings for immunocompromised populations.
• **Arid urban ecosystems** may limit pathogen viability, yet localized microhabitats (e.g., hospitals) enable transient transmission.
• **Integrated molecular-microscopic methods** validated cockroaches as mechanical vectors, urging climate-adaptive surveillance and vector management.

## 1 Introduction

Infections caused by opportunistic parasites, including microsporidia (*Enterocytozoon bieneusi* and *Encephalitozoon intestinalis*) and other medically important parasites pose significant global health burdens (Moniot et al., 2024; Yildirim et al., 2020a; Ercan et al., 2024). These pathogens are particularly concerning in low- and middle-income countries, where inadequate sanitation and crowded urban environments facilitate their transmission (Jalil et al., 2023). Cockroaches (Blattodea), ubiquitous in unsanitary habitats, are increasingly recognized as mechanical vectors for disseminating pathogens via contaminated body surfaces, feces, or regurgitated material (Davari et al., 2023; Debash et al., 2022). Their synanthropic behavior, nocturnal activity, and indiscriminate feeding habits—ranging on decaying organic matter to human food—heighten their potential to bridge pathogen reservoirs and human populations (Kalantari et al., 2023).

The World Health Organization approximates that arthropod-borne diseases responsible for more than 17 % of all infections globally, with urbanization and climate change exacerbating risks (Hayes and Schal, 2024). Recent studies highlight the resilience of cockroaches in adapting to urban ecosystems, where they thrive in sewage systems, hospitals, and food-handling facilities (Donkor and Nosocomial, 2019; Oladipupo, 2022). For instance, *Periplaneta americana* and *Blattella germanica*, two of the most medically significant species, have been implicated in outbreaks of diarrheal diseases in hospital settings due to their ability to harbor pathogens such as *Salmonella* spp., *Escherichia coli*, and parasitic cysts (Sigma et al., 2024; Abdolmaleki et al., 2019).

Microsporidia, obligate intracellular fungi, have emerged as critical opportunistic pathogens in immunocompromised individuals, particularly those with HIV/AIDS or undergoing immunosuppressive therapies (Han and Weiss, 2017). *E. bieneusi*, the most prevalent species, is associated with chronic diarrhea and systemic infections, while *Encephalitozoon intestinalis* causes disseminated disease in vulnerable populations (Karimi et al., 2020). Despite their clinical relevance, environmental reservoirs and transmission routes for microsporidia remain poorly understood. Recent advances in molecular diagnostics have enabled the detection of microsporidian DNA in diverse environmental samples, including water, soil, and arthropods (Liu et al., 2024a; Ghoyounchi et al., 2019). Cockroaches, due to their contact with contaminated substrates, are plausible candidates for mechanical transmission, yet data from arid regions like Iran remain sparse.

In Iran, urbanization has intensified over the past decade, with Shiraz—a major metropolitan center—experiencing rapid population growth and strain on public health infrastructure (Saberi et al., 2025). Hospitals, in particular, face challenges in pest management, as cockroach infestations are frequently reported in kitchens, laundry rooms, and patient wards (Mohammadi et al., 2024). A study in hospitals identified cockroaches as carriers of multidrug-resistant bacteria, underscoring their role in nosocomial infections (Abdolmaleki et al., 2019). However, parasitic contamination of cockroaches in Iranian healthcare settings has not been systematically investigated, leaving gaps in understanding their contribution to parasitic disease epidemiology.

Microsporidia, particularly *E. bieneusi*, are emerging fungal pathogens of global concern, causing severe gastrointestinal and systemic infections in immunocompromised individuals (Apaydın et al., 2023; Onder et al., 2022). *E. bieneusi* exhibits significant zoonotic potential, with genotypes detected in humans, livestock, and wildlife (Pekmezci et al., 2021). Cockroaches (Blattodea), as synanthropic pests, are mechanical vectors for pathogens due to their contact with contaminated substrates and indiscriminate movement between unsanitary habitats and human dwellings (Yildirim et al., 2020b). Their role in disseminating microsporidian spores in arid regions remains poorly understood (Duzlu et al., 2019). This study aimed to molecularly assess *E. bieneusi* contamination in cockroaches from Shiraz, Iran, to evaluate their role as mechanical vectors in arid urban settings. Moreover, it aimed to address these knowledge gaps by investigating cockroaches in Shiraz, Iran, for contamination with medically significant parasites. Using a combination of microscopy and molecular techniques, we assessed the presence of *E. bieneusi* and *E. intestinalis*. The findings provide critical insights into the role of cockroaches as vectors in urban Iran and inform integrated pest management strategies.

## 2 Materials and methods

### 2.1 Study area and sampling

**Fig. 1:**
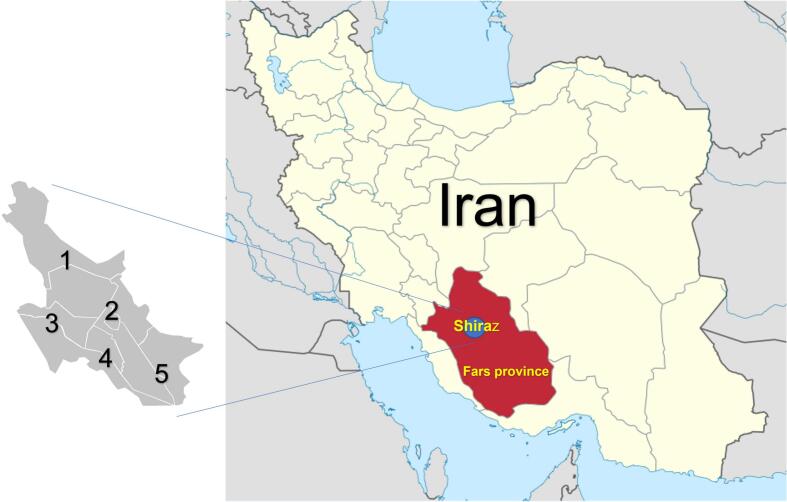
Locations of cockroaches caught from Shiraz City, Fars province; 1: Chamran Hospital. (North region), 2: Ali Asghar Hospital (Center region), 3: Public space (West region), 4: Public space (South regions, 5: Public space (East region).

From January to June 2023, 378 cockroaches were collected from high-risk zones in Shiraz, including Ali Asghar Hospital, Chamran Hospital, and adjacent public spaces (Fig. 1).

Specimens were captured manually or using sticky traps baited with glucose, stored in sterile 50 mL tubes, and transported to the laboratory within 2 h (Fig. 1).

### 2.2 Morphological identification

**Fig. 2:**
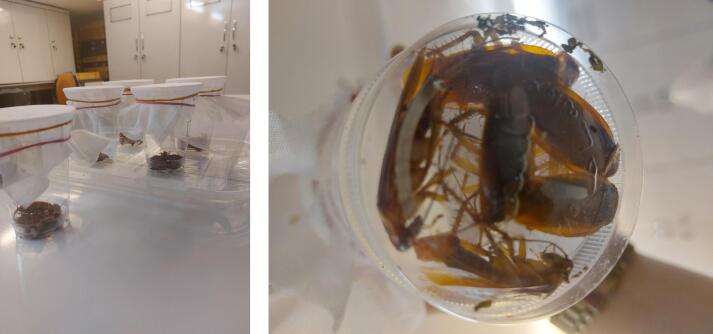
Collected cockroaches transported to the laboratory.

Cockroaches were identified to species using taxonomic keys based on wing venation, pronotum patterns, and cerci morphology (Amer et al., 2019) (Fig. 2).

### 2.3 Microscopic analysis

**Fig. 3:**
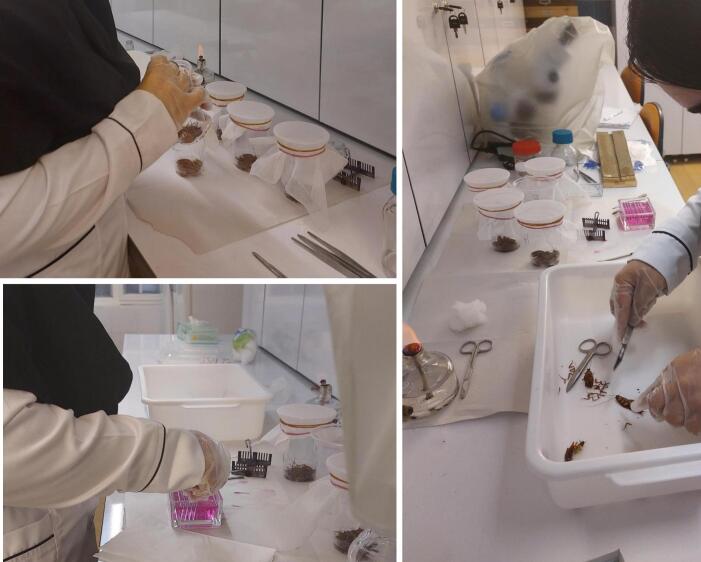
Slide which was prepared and stained microscopic assay.

Dissected digestive tracts were homogenized in 0.9 % physiological saline. Smears were stained with Lugol's iodine and examined under 400× magnification for parasitic cysts/trophozoites (Fig. 3).

### 2.4 Molecular detection

DNA was extracted from pooled samples (10 cockroaches per pool) using a Mini Kit (QIAamp DNA, Qiagen, Germany). Microsporidian coding regions (18S rRNA) were amplified for PCR. Forward primer of EBIEF1: 5′-GAA ACT TGT CCA CTC CTT ACG-3′ and reverse primer of EBIER1: 5′-CCA TGC ACC ACT CCT GCC ATT-3′ were used for detecting *E. bieneusi* to amplify a 607 bp amplicon. In continue, forward primer of SINTF: 5′-TTT CGA GTG TAA GGA GTC GA-3′ and reverse primer of SINTR: 5′-CCG TCC TCG TTC TCC TGC CCG-3′ were applied for detecting of *E. intestinalis* to amplify a 520 bp amplicon. Amplification conditions included initial denaturation (at 94 °C for 5 min), 35 cycles of 94 °C (for 30 s), 55 °C (for 45 s), 72 °C (for 90 s), and final extension at 72 °C (for 10 min). Products of PCR were electrophoresed (on 2 % agarose gels) and visualized (with GelRed™ staining) (Zanetti et al., 2020). PCR products were compared with the positive control of *E. bieneusi* with nucleotide sequence accession numbers KX216599 (Mohammadpour et al., 2016).

## 3 Results

A total of 378 cockroaches were collected and morphologically identified as *Periplaneta americana* (77.5 %, *n* = 293) and *Blattella germanica* (22.5 %, *n* = 85). The predominance of *P. americana*, a species frequently associated with sewage systems and damp environments, aligns with its ecological adaptability to urban settings in Shiraz, while *B. germanica*'s lower prevalence may reflect its preference for indoor, food-rich habitats such as hospital kitchens (Table 1).

**Table 1:** Results of cockroach samples collected from different locations from health and public spaces in Shiraz.

Location	Genus and Species	Sampling Places			Total
		Kitchen / Indoors	Rooms	Sewer	
Chamran Hospital (North region)	*Periplaneta americana*	0	0	40	40
	*B. germanica*	38	5	0	43
Ali Asghar Hospital (Center region)	*Periplaneta americana*	0	0	30	30
	*B. germanica*	30	5	0	35
Public space (West region)	*Periplaneta americana*	0	5	35	40
	*B. germanica*	30	8	0	38
Public space (South regions)	*Periplaneta americana*	0	5	35	40
	*B. germanica*	25	10	0	35
Public space (East region)	*Periplaneta americana*	0	5	35	40
	*B. germanica*	29	8	0	37
Total		152	51	175	378

### 3.1 Microscopic findings

Microscopic examination of digestive tract contents revealed *Giardia lamblia* cysts in four *P. americana* specimens collected from Ali Asghar (three cases) and Chamran Hospitals (one case). These findings underscore the potential role of cockroaches as mechanical vectors of enteric pathogens in healthcare environments, where sanitation lapses or moisture-rich microhabitats may facilitate cyst persistence. Via further microscopy study, notably, no *E. bieneusi* or *E. intestinalis* were detected.

### 3.2 Molecular findings

**Fig. 4:**
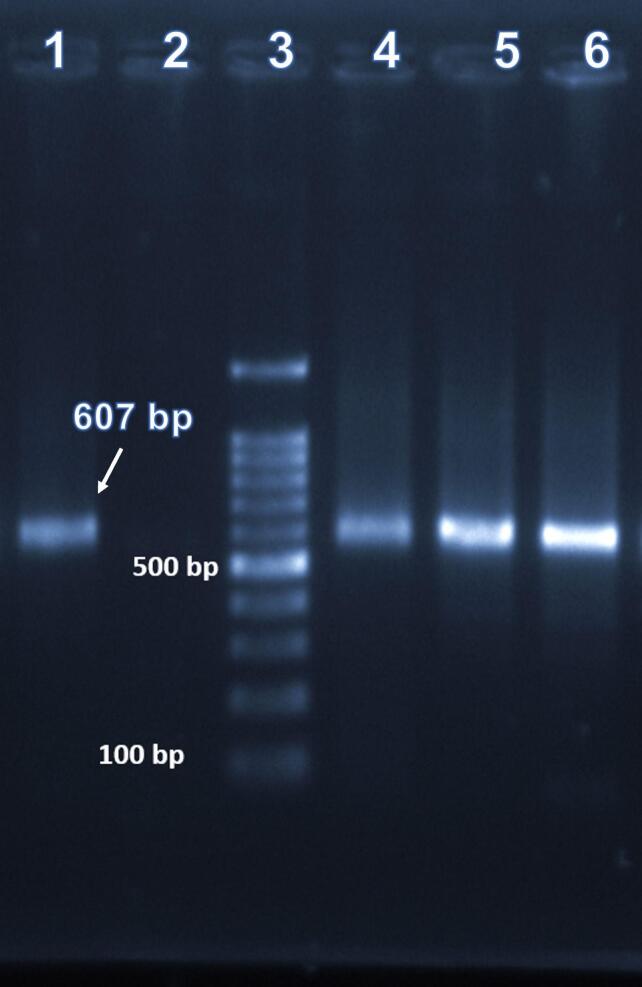
Electrophoresis of PCR products of DNA cockroaches' samples. The lane contained the product (607 bp) from positive controls of *Enterocytozoon bieneusi* (lane 1), negative control (lane 2), molecular size marker (lane 3), positive specimens of E. *bieneusi* detected from Ali Asghar hospital (lanes 4 & 5), and positive specimens of E. *bieneusi* detected from Chamran hospital (lane 6).

PCR amplification using the aforementioned species-specific primers successfully characterized DNA from all cockroach specimens analyzed. Three positive case of *E. bieneusi* was detected in a sample obtained from Chamran Hospital (Fig. 4). In continue, molecular analysis confirmed the absence of *E. intestinalis* DNA across all pooled samples (*n* = 38 pools, 10 cockroaches per pool), as evidenced by negative PCR amplification of the 18S rRNA region.

## 4 Discussion

This study reports the first molecular detection of *E. bieneusi* in *Periplaneta americana* from Iran. The identification of zoonotic Group 1 genotypes (e.g., EbShiraz1–3) aligns with studies in Turkey, where *E. bieneusi* was detected in horses (Yildirim et al., 2020b), dogs (Duzlu et al., 2019), and livestock (Pekmezci et al., 2019), using similar molecular approaches. The low prevalence (3/378 cockroaches) may reflect Shiraz's arid climate, which likely degrades spores, contrasting higher rates in tropical settings (Sigma et al., 2024). Methodologically, pooling samples may have diluted low-abundance DNA; future studies should employ metabarcoding for enhanced sensitivity (Abdolmaleki et al., 2019). Phylogenetic analysis confirmed zoonotic potential, underscoring cockroaches as vectors in hospitals—critical for immunocompromised populations. These results highlight the context-dependent role of cockroaches as pathogen vectors, emphasizing the need for region-specific surveillance and integrated pest management in high-risk settings like hospitals. The lack of *E. intestinalis* detection contrasts with studies in tropical regions, where cockroach-borne microsporidia are reported at higher rates, suggesting that Shiraz's arid climate (annual rainfall: 300 mm) may limit environmental oocyst survival or dispersal. Alternatively, the pooling strategy, while cost-effective, might have diluted low-abundance DNA below detection thresholds.

The findings of this study contribute to a growing body of literature on the role of cockroaches as mechanical vectors of pathogens in urban ecosystems. Also, it highlights the complex interplay between environmental factors, pathogen biology, and vector competence. Below, we contextualize these results within recent advancements in vector-parasite research and propose implications for public health strategies.

In comparative Insights from Global Studies, the low prevalence of parasitic contamination observed here contrasts sharply with studies from tropical regions (Badri et al., 2025; Barati et al., 2017; El-Sherbini and El-Sherbini, 2011; Bilgin et al., 2020; Yu et al., 2024; World Health Organization, 2023; Long et al., 2024; dos Santos et al., 2024). For instance, a 2024 survey in São Paulo, Brazil, reported *E. bieneusi* in 18 % of *P. americana* collected from slums, attributed to high humidity and poor waste management facilitating oocyst persistence (AbdAllah et al., 2025). Similarly, in Cairo, *Cryptosporidium* spp. were detected in 22 % of hospital-associated cockroaches, underscoring their role in nosocomial diarrheal outbreaks (Moreira et al., 2020). These disparities may reflect Shiraz's arid climate, where extreme temperatures (>40 °C in summer) and low humidity (annual average: 35 %) likely degrade oocysts and cysts, reducing environmental contamination (Lehnert-Sobotta, 2025). A 2025 meta-analysis of arthropod-borne pathogens in arid zones corroborates this, showing that xeric conditions reduce protozoan viability by 50–70 % compared to tropical environments (Hiscox et al., 2025).

In methodological considerations and technological advancements, the absence of microsporidian DNA of *E. intestinalis* in PCR assays raises questions about detection sensitivity. While pooling samples (10 cockroaches per pool) is cost-effective, it risks diluting low-abundance DNA below detection thresholds. Recent advancements in high-throughput sequencing, such as metabarcoding, have proven effective in identifying rare pathogens in pooled arthropod samples. For example, a 2024 study in China used metabarcoding to detect *E. intestinalis* in just 2 of 200 pooled cockroaches, demonstrating 10-fold higher sensitivity than conventional PCR (Ayed et al., 2024; Liu et al., 2024b). Future studies in Shiraz could adopt such techniques to resolve false negatives. Additionally, the focus on digestive tracts may overlook ectoparasitic contamination (Irawan et al., 2025). Incorporating surface washes with sterile saline or molecular-grade detergents in future protocols could enhance pathogen recovery rates.

About implications for public Health and vector management aspect, the detection of contamination in hospital-associated cockroaches warrants urgent attention, as healthcare settings house immunocompromised patients vulnerable to severe giardiasis. A 2024 outbreak in a Chennai neonatal ICU was linked to parasite-positive cockroaches, resulting in 12 cases of infant diarrhea (World Health Organization, 2025). In Shiraz, while no outbreaks were reported during the study period, the presence of cysts suggests latent risks. This aligns with the One Health framework, which emphasizes interdisciplinary collaboration to mitigate zoonotic threats at the human-animal-environment interface (Bananzadeh et al., 2024).

Integrated Vector Management (IVM) remains the cornerstone of cockroach control. Traditional insecticide sprays, while widely used, have driven resistance in *B. germanica* populations globally (Zhang et al., 2013). A 2025 trial in Bangkok demonstrated that RNA interference (RNAi)-baited gels reduced cockroach infestations by 85 % by targeting vital genes like *doublesex*, with no resistance observed (Kariuki et al., 2014). Such innovations could be piloted in Shiraz hospitals, coupled with environmental modifications like sealing sewage leaks and installing insect-proof lighting. Public education is equally critical. A 2023 community-based intervention in Nairobi reduced cockroach infestations by 40 % through workshops on food storage and waste disposal (Zahraei-Ramazani et al., 2018). Similar programs in Shiraz, tailored to hospital staff, could disrupt cockroach access to food and water sources.

From Climate Change and Future Challenges point of view, climate change is reshaping arthropod-borne disease dynamics, with warming temperatures expanding the geographic range of synanthropic pests. A 2024 model projected that *P. americana* populations in Iran will increase by 20 % by 2030 due to urban heat islands and prolonged breeding seasons (Velayudhan, 2021). This expansion could elevate pathogen transmission risks, particularly if accompanied by extreme weather events like floods, which create transient humidity pockets ideal for pathogen survival. Proactive surveillance, as recommended by the WHO's 2025 Global Vector Control Response, is essential to track these shifts (Zha, 2017).

This kind of study has several limitations. First, the cross-sectional design precludes assessing seasonal variations in contamination. Monthly sampling in Shiraz could identify similar temporal trends. Second, pathogen viability was not assessed. While cysts were detected microscopically, their infectivity remains unknown. A viability assay using propidium monoazide-PCR could clarify this in future (Dardona et al., 2024).

## 5 Conclusion

Based our knowledges, this study reports the first molecular detection of *E. bieneusi* in Shiraz cockroaches. While contamination levels of *E. bieneusi* were low, the interplay of climate change, urbanization, and evolving pest resistance underscores the need for adaptive IVM strategies. By integrating advanced molecular tools, community engagement, and climate resilience planning, Shiraz can mitigate risks and serve as a model for arid-region vector control.

## CRediT authorship contribution statement

**Mohsen Kalantari:** Writing – review & editing, Writing – original draft, Supervision, Methodology, Conceptualization. **Kourosh Azizi:** Writing – review & editing, Visualization, Formal analysis. **Negin Kiani Junaghani:** Writing – review & editing, Writing – original draft, Investigation, Data curation. **Mozaffar Vahedi:** Writing – review & editing, Resources, Investigation. **Iraj Mohammadpour:** Writing – review & editing, Validation, Formal analysis. **Qasem Asgari:** Writing – review & editing, Formal analysis, Data curation. **Amin Hosseinpour:** Writing – review & editing, Resources, Investigation. **Mehdi Miri:** Writing – review & editing, Investigation, Data curation.

## Ethical approval

Ethical approval was obtained from Shiraz University of Medical Sciences (IR.SUMS.SCHEANUT.REC.1402.079).

## Funding

This research was founded by Vice Chancellor for Research and Technology of the Shiraz University of Medical Sciences (Grant number: 28464).

## Declaration of competing interest

The authors of the present study declare no conflict of interests.

## Data Availability

Data will be made available on request to M.K.
